# *MYBPC3* mutations are associated with a reduced super-relaxed state in patients with hypertrophic cardiomyopathy

**DOI:** 10.1371/journal.pone.0180064

**Published:** 2017-06-28

**Authors:** James W. McNamara, Amy Li, Sean Lal, J. Martijn Bos, Samantha P. Harris, Jolanda van der Velden, Michael J. Ackerman, Roger Cooke, Cristobal G. dos Remedios

**Affiliations:** 1 Discipline of Anatomy & Histology, Bosch Institute, University of Sydney, Sydney, NSW, Australia; 2 Division of Cardiovascular Health and Disease, University of Cincinnati College of Medicine, Cincinnati, OH, United States of America; 3 Department of Molecular Physiology and Biophysics, University of Vermont, Burlington, VT, United States of America; 4 Department of Pediatric and Adolescent Medicine, Division of Pediatric Cardiology, Mayo Clinic, Rochester, MN, United States of America; 5 Department of Molecular Pharmacology & Experimental Therapeutics Windland Smith Rice Sudden Death Genomics Laboratory, Mayo Clinic, Rochester, MN, United States of America; 6 Department of Cellular and Molecular Medicine, University of Arizona, Tucson, AZ, United States of America; 7 Department of Physiology, Institute for Cardiovascular Research, VU University Medical Center, De Boelelaan 1117, Amsterdam, The Netherlands; 8 Department of Cardiovascular Diseases, Division of Heart Rhythm Services, Mayo Clinic, Rochester, MN, United States of America; 9 Department of Biochemistry and Biophysics, Cardiovascular Research Institute, University of California, San Francisco, CA, United States of America; Semmelweis Egyetem, HUNGARY

## Abstract

The “super-relaxed state” (SRX) of myosin represents a ‘reserve’ of motors in the heart. Myosin heads in the SRX are bound to the thick filament and have a very low ATPase rate. Changes in the SRX are likely to modulate cardiac contractility. We previously demonstrated that the SRX is significantly reduced in mouse cardiomyocytes lacking cardiac myosin binding protein–C (cMyBP-C). Here, we report the effect of mutations in the cMyBP-C gene (*MYBPC3*) using samples from human patients with hypertrophic cardiomyopathy (HCM). Left ventricular (LV) samples from 11 HCM patients were obtained following myectomy surgery to relieve LV outflow tract obstruction. HCM samples were genotyped as either *MYBPC3* mutation positive (*MYBPC3*_mut_) or negative (HCM_smn_) and were compared to eight non-failing donor hearts. Compared to donors, only *MYBPC3*_mut_ samples display a significantly diminished SRX, characterised by a decrease in both the number of myosin heads in the SRX and the lifetime of ATP turnover. These changes were not observed in HCM_smn_ samples. There was a positive correlation (p < 0.01) between the expression of cMyBP-C and the proportion of myosin heads in the SRX state, suggesting cMyBP-C modulates and maintains the SRX. Phosphorylation of the myosin regulatory light chain in *MYBPC3*_mut_ samples was significantly decreased compared to the other groups, suggesting a potential mechanism to compensate for the diminished SRX. We conclude that by altering both contractility and sarcomeric energy requirements, a reduced SRX may be an important disease mechanism in patients with *MYBPC3* mutations.

## Introduction

Hypertrophic cardiomyopathy (HCM) is an inherited disorder affecting more than 1 in 500 people worldwide [1]. It is characterised by an enlarged heart, thickened left ventricular (LV) walls (>15 mm) and reduced chamber capacity in the absence of other causes such as hypertension or aortic stenosis [2]. Commonly, HCM patients exhibit enhanced systolic function, while diastolic function is often impaired at an early stage of the disease. HCM is also associated with an increased risk of arrhythmias and sudden cardiac death, particularly in younger patients [3]. Genetic mutations that result in defective sarcomeric proteins are the most common cause of HCM [4–6]. In particular, mutations in the genes that encode the thick filament proteins cardiac myosin binding protein-C (*MYBPC3*) and β-myosin heavy chain (*MYH7*) account for about 80% of all genotype positive HCM cases [7].

**Fig 1:**
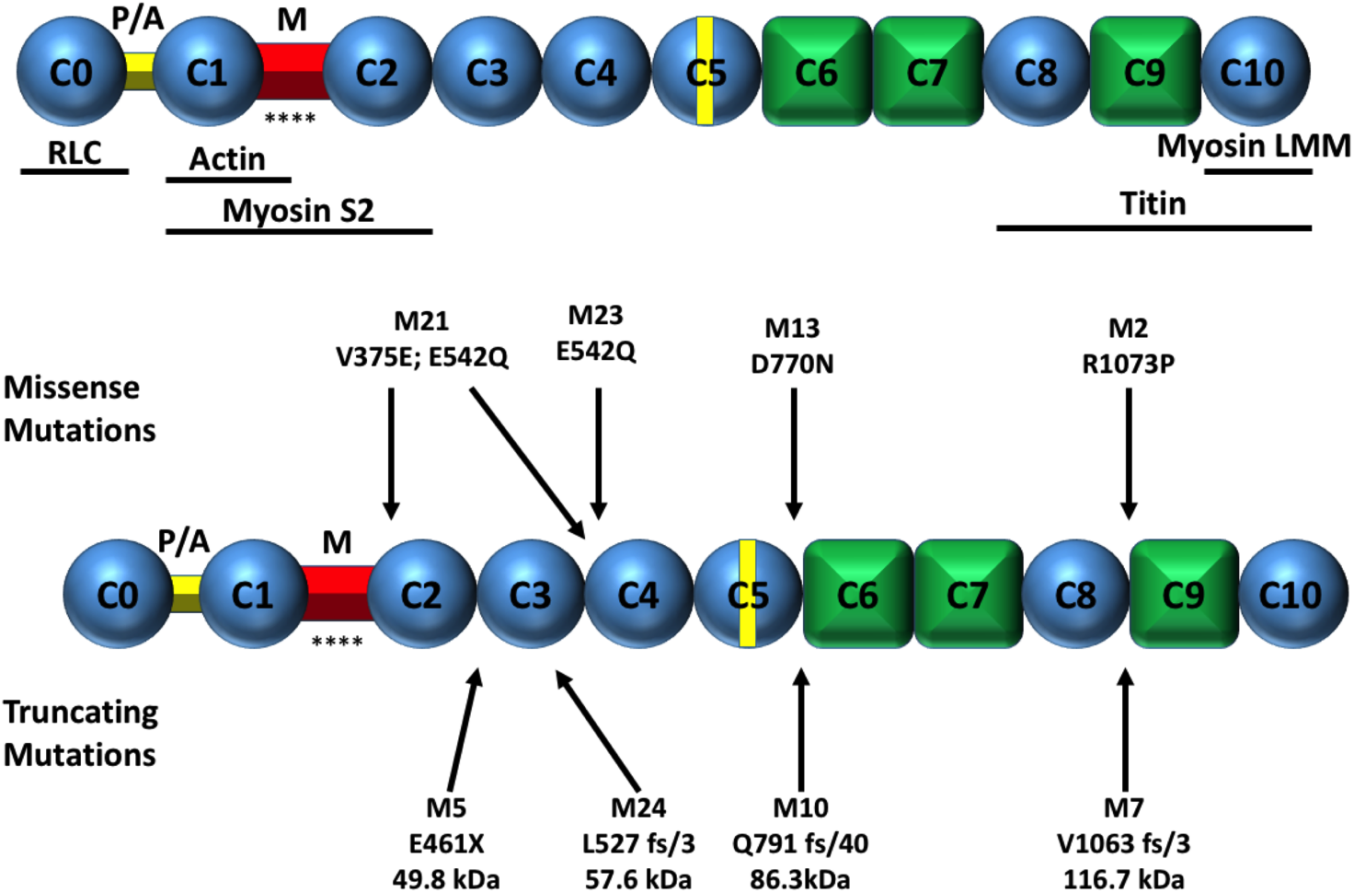
Schematic of the basic structure of cMyBP-C. **A** Diagram showing the domain structure of cMyBP-C. Ovals represent Ig-like domains and diamonds represent FnIII-like domains. P/A is the pro/ala region and M the phosphorylatable domain (* represents phosphorylatable serine sites within this domain). Regions of cMyBP-C that interact with other sarcomeric proteins are shown below this. **B** cMyBP-C missense and truncation samples used in this study are shown above and below the schematic, respectively.

cMyBP-C is a thick filament associated protein with a molecular weight of ~150 kDa, first identified as a contaminant of myosin preparations [8]. It is the protein product resulting from the *MYBPC3* gene. It localises as 7–9 transverse stripes within the inner two thirds of each half A-band (termed the C-zone) [9] and is exclusively expressed in the heart. However, the two skeletal isoforms (encoded by separate genes) may also be conditionally present [10]. cMyBP-C consists of 11 major domains named C0-C10, eight of which are immunoglobulin-like domains while the remaining three are fibronectin-like domains (Fig 1). The N-terminal linker regions consist of the proline-alanine rich region located between C0 and C1 [11], and the phosphorylatable M domain between the C1 and C2 domains [12]. The C-terminal domains of cMyBP-C binds to the light meromyosin (LMM) portion of β-myosin heavy chain [13] and to titin [14]. Residues in the N-terminus can interact with actin [15], the S1/S2 junction of myosin heavy chain [13], and the myosin regulatory light chain (RLC) [16]. These multiple N-terminal interactions have been shown to regulate cardiac contractility [17–19].

The “super-relaxed state” (SRX) of myosin is characterised by a strongly inhibited ATPase activity [20–24]. While this inhibited state of myosin was predicted nearly 40 years ago [25], it is still perceived as an emerging area in striated muscle research (reviewed in [26, 27]). Myosin heads in the SRX are aligned along the thick filament surface in a quasihelical manner [28], restricting their interaction with actin. This arrangement (termed the “interacting heads motif”) is analogous to the “OFF” state in smooth muscle myosin [29], and has been reported in striated muscle from a range of evolutionarily distinct species, including human left ventricle [30–33]. The SRX is in equilibrium with another state of myosin, defined as the disordered relaxed state (DRX). Myosin heads in the DRX are not immobilised on the thick filaments and may weakly bind actin. The ATPase activity of DRX myosin is ~10 times faster than SRX myosin [34]. Therefore, in cardiac muscle, the relative populations of these two states determines both the number of myosin heads available for contraction and the energetic requirements of the sarcomere.

We recently reported a severe destabilisation of the SRX in mouse LV muscle lacking cMyBP-C [22]. However, while these mice lacked the *MYBPC3* gene, heterozygous mice developed no disease [35]. While this provides insight into the role of cMyBP-C in the SRX, it is not necessarily representative of *MYBPC3* mutations in patients with HCM, since they are almost exclusively heterozygous. In order to better understand the effect of these human *MYBPC3* mutations, we measured the SRX in skinned myocytes obtained from humans diagnosed with HCM. *Compared to explanted*, *non-diseased donor hearts*, *we found that only myocytes with MYBPC3 mutations—and not those without a sarcomeric mutation–exhibit a disturbed SRX in HCM samples*.

## Methods

### Cardiac sample collection and ethics

Cardiac tissue from 11 HCM patients was obtained from the LV interventricular septum during myectomy surgery aimed to relieve LV outflow tract obstruction. Non-failing cardiac tissue was obtained from donor hearts where no suitable transplant recipient was found at the time, to serve as healthy, non-HCM controls. It should be noted that while no formal data exist for the ejection fractions of these donors, it is a requirement for donation that ventricular function is normal, based on a bedside echocardiogram at the time of organ harvest. These donor hearts have been extensively studied [36–42]. For donor hearts, LV tissue was used. Donor hearts used in this study had been collected between 1997 to 2013. Donor hearts were flushed with ice-cold cardioplegic solution, packaged under sterile conditions, and transported within four hours to the Sydney Heart Bank, Bosch Institute by with the assistance of the Australian Red Cross Blood Service. No donors were from a vulnerable population, and informed consent was obtained for each sample prior to tissue procuration. All patient data was accessed anonymously. All samples were immediately flash-frozen and stored in liquid nitrogen dewars until required. Table 1 summarises patient data for all the samples used in this study.

**Table 1:** Patient data of samples used in this study. For expanded HCM patient data, see S1 and S2 Tables.

*MYBPC3*_mut_	M2	54	M	Missense	R1073W	-	42	67	Still Living
	M21	15	M	Missense	E542Q	*MYBPC3* V375E	N/A		Still Living
								67	
	M23	54	F	Missense	E542Q	-	47	75	Unknown
	M13	38	F	Missense	D770N	*MYL3* E143K	39	67	Still Living
	M5	23	F	Truncation	E461X	-	44	71	Still Living
	M24	54	F	Truncation	L527 fs/3	-	44	72	Still Living
	M10	45	M	Truncation	Q791 fs/40	-	38	69	Still Living
	M7	30	F	Truncation	V1063 fs/63	-	49	76	Still Living
Non-Failing Donors	2.158	17	M	N/A	N/A		N/A	-	Head Injury
	3.168	19	F	N/A	N/A		N/A	-	Motor vehicle accident
	5.138	23	M	N/A	N/A		N/A	-	Hypoxic Brain Injury
	6.008	40	M	N/A	N/A		N/A	-	Middle Cerebral Artery Haemorrhage
	8.01	43	F	N/A	N/A		NA	60%	Middle Cerebral Artery Haemorrhage
	3.164	61	M	N/A	N/A		N/A	-	N/A
	6.076	62	F	N/A	N/A		NA	-	Hypoxic Brain Injury
	7.028	62	M	N/A	N/A		N/A	-	Middle Cerebral Artery Haemorrhage
HCM_smn_	A48	49	M	0/9	N/A		41		Unknown
	A45	52	M	0/9	N/A		52		Unknown
	A22	53	M	0/9	N/A		56		Unknown

This study was approved by and adheres to the University of Sydney Human Research Ethics Committee (HREC) approval #2012/2814. Myectomy samples from the Mayo Clinic were obtained with the approval of the Mayo Clinic Human Ethics Committee following approval by its Institutional Review Board (IRB) (#811-98-22) and transferred to the University of Sydney occurred under HREC approval (09-2009/12146). Transfer of those samples from The Netherlands occurred under a Material Transfer Agreement with the University of Sydney Human Research Ethics Committee (2012/030).

### Sample genotyping

Myectomy samples from the Mayo Clinic were obtained at the time of surgery and immediately flash frozen and stored in liquid nitrogen. Comprehensive mutational analysis was performed using denaturing high performance liquid chromatography and direct DNA sequencing for the nine most common HCM-associated sarcomeric genes (*MYH7*, *MYBPC3*, *MYL2*, *TNNC3*, *TNNT2*, TNNI3, *TPM1*, *MLP* and *ACTC1*). Patients with *MYBPC3* mutations (*MYBPC3*_mut_) were selected for this study. A separate group of myectomy samples was obtained from The Netherlands. Genotyping of these samples was negative for mutations in the above nine most commonly mutated sarcomeric genes. These sarcomeric mutation negative samples (HCM_smn_) allowed us to investigate functional differences due to mutations in *MYBPC3* or cardiac hypertrophy associated with HCM.

### SRX solutions

The SRX solutions used have been described previously [22]. Cardiac muscle was skinned in a buffer with pH 7, which contained (in mM): 100 NaCl, 8 MgCl_2_, 5 EGTA, 5 each of K_2_HPO_4_ and KH_2_PO_4_, 3 NaN_3_, 5 ATP, 1 DTT, 20 2,3-butanedione monoxime (BDM), and 0.01% (v/v) Triton-X 100. Following skinning, muscle was stored in a 50% (v/v) glycerol buffer at pH 6.8, consisting of (in mM): 120 K acetate, 5 Mg acetate, 2.5 each of K_2_HPO_4_ and KH_2_PO_4_, 5 EGTA, 50 MOPS, 5 ATP, 20 BDM, and 2 DTT.

The rigor buffer was prepared at pH 6.8 and consisted of (in mM): 120 K acetate, 5 Mg acetate, 2.5 each of K_2_HPO_4_ and KH_2_PO_4_, 5 EGTA, 50 MOPS and 2 DTT. DTT was added fresh each day. The relaxing solution was identical to the rigor buffer, but also contained 4 mM ATP. Similarly, the mATP buffer was identical to the rigor buffer, but contained 250 μM of the fluorescent nucleotide 2′-/3′-O-(N′-methylanthraniloyl)adenosine-5′ -O triphosphate (mATP; Sapphire Bioscience, Cat # NU-202L).

### Preparation of permeabilised cardiomyocytes

Detergent skinned muscle was prepared as previously described [22]. Briefly, ~10 mg of cryopreserved LV was obtained under liquid nitrogen and immersed in skinning solution on ice. Samples were incubated for 6 hours with gentle agitation and the solution refreshed twice. Glycerinating buffer then replaced the skinning solution and the samples were equilibrated overnight on ice (~15 h). The glycerinating solution was refreshed and samples stored at -20°C for use up to 1 week.

### SRX experiments and analysis

Measurements of the SRX were carried out as previously described [22]. Briefly, small bundles of detergent skinned muscle cells, length ~1-2mm, diameter ~60–80μm, were hand dissected under a stereomicroscope at 4°C. Using two layers of double-sided adhesive tape, cardiomyocytes were immobilised on the surface of a glass coverslip and mounted onto a cooled glass slide, creating a flow cell. An illustration of this apparatus is presented in Figure B in S1 Text. Glycerinating solution was flushed through the flow cell and the slides stored on ice until imaging within a few hours.

Prior to imaging, ATP, BDM and glycerol were removed by multiple washes with rigor buffer. Cardiomyocytes were then incubated in the fluorescent mATP buffer for at least 5 min before imaging. Imaging was carried out using a 20x (0.75 NA) objective on an upright Nikon Ni-E epifluorescence microscope, with a Luencor SpectraX LED (excitation 395 nm, emission wavelength 460 nm bandpass filter, exposure time 20 ms). Images were acquired every 5 s using a DS-Qi2 monochrome camera. After imaging for 60 s, mATP buffer was chased over a 10 s period with ATP buffer to ensure diffusion of nucleotides out of the muscle bundles [43]. It is important to note that these assays are done on completely relaxed tissue, due to the Ca^2+^ chelating effect of 5 mM EGTA, allowing the structure of the thick filament to be studied without the confounding factor of thin filament activation.

For analysis, two rectangular regions-of-interest (~15 x 30μm) were drawn over both the fibre and the background adjacent to the fibre. The fluorescence intensity of the background was subtracted from the fibre. Fluorescence intensity (I) of the tissue was normalised to the value immediately before washout of mATP with ATP, and the resulting curve fit to the equation:

I=1−P1(1−exp⁡(−tT1))−P2(1−exp⁡(−tT2))

Where P1 and P2 are the relative proportions of fluorescence attributed to each state, t is the time in the ATP chase, and T1 and T2 are the time constants for their respective states. For complete interpretation of the equation, see [22]. Non-linear regression by the least squares was carried out using GraphPad Prism 6.0 using a custom made two-phase decay equation (described above). Initial values for the four parameters were set as P1 = 0.7, T1 = 13s, P2 = 0.3, T2 = 120s.

### Protein isolation

In order to measure the endogenous phosphorylation of sarcomeric proteins and expression of cMyBP-C, samples were treated with trichloroacetic acid (TCA) using a similar method as previously published [44]. Briefly, a ~20 mg piece of tissue taken adjacent from the tissue used for SRX experiments was homogenised in a liquid nitrogen cooled mortar and pestle, then mixed with 1 ml of TCA solution, consisting of10% w/v TCA in acetone, containing 0.1% w/v DTT). Samples were then gradually warmed to room temperature with steps at -80°C (1 hr), -20°C (20 min), 4°C (20 min) and room temperature (20 min) with vortexing between each step. Samples were then centrifuged at 12,000xg for 5 min and the TCA solution replaced with 1 ml of acetone washing solution of 100% acetone containing 0.2% w/v DTT, and shaken for 5 min. The acetone wash was repeated twice. The acetone solution was then discarded and the pellet allowed to air dry before adding ~150 μl of pellet solubilisation buffer, which contained 8 M urea, 50 mM tris-HCl pH 6.8, 2% SDS, 10% glycerol, and 1 mM β-mercaptoethanol. Samples were heated at 95°C for 5 min and the pellet dispersed using a manual homogeniser followed by incubation at 4°C for 1 hour with shaking. Samples were spun on a bench-top centrifuge briefly to pellet any insoluble debris and the supernatant was collected and stored at -80°C. Protein concentration was determined by the BCA assay (Thermo-Fisher) [45].

#### ProQ diamond and Sypro Ruby staining

Endogenous phosphorylation was measured by ProQ Diamond stained gels, in a manner similar to [44]. 35 μg of protein was separated on hand cast 12% SDS-PAGE gels. Gels were run at 80 V for the first 30 min, and then at 120 V until the tracking dye reached the bottom of the gel (~90 min). Following electrophoresis, gels were fixed overnight, stained with ProQ diamond (90 min and turned 90° after 45 min) and destained (3 x 30 min) according to the manufacturers guidelines. Imaging was carried out using the Bio-Rad ChemiDoc imaging system with ProQ diamond default settings (green epi illumination, 605/50 filter). After imaging, the gel was stained with Sypro Ruby (overnight) to measure total protein content. Imaging was carried out with the Sypro Ruby settings (UV trans illumination, 605/50 filter). A Peppermint Stick (ThermoFisher) molecular weight marker was used for molecular weight estimation. This ladder contains a mixture of phosphorylated and dephosphorylated molecular weight markers, acting as both positive and negative controls for the staining process.

#### Measurement of relative cMyBP-C content

20 μg of protein was separated on 4–15% gradient stain-free SDS-PAGE gels (Bio-Rad TGX stain free gels). Proteins were transferred to PVDF membranes using semi-dry blotting (Bio-Rad). Expression levels of cMyBP-C were measured using a specific antibody to cMyBP-C (HPA043898, 1:2000 dilution), and detected with a HRP-conjugated goat anti-rabbit secondary antibody (Sigma-Aldrich A0545, 1:7500 dilution). Bands were detected with ECL Prime (GE Healthcare) using the Bio-Rad ChemiDoc system. The expression of cMyBP-C was normalised to α-actinin as reported previously [42].

### Statistical analysis

All data are represented as means ± S.E.Ms. Differences between groups were assessed using one-way ANOVA (Prism 6.0) followed by a Tukey post-hoc analysis with significance accepted at p < 0.05.

## Results

### Presence of a super-relaxed state (SRX) in the human left ventricle

**Fig 2:**
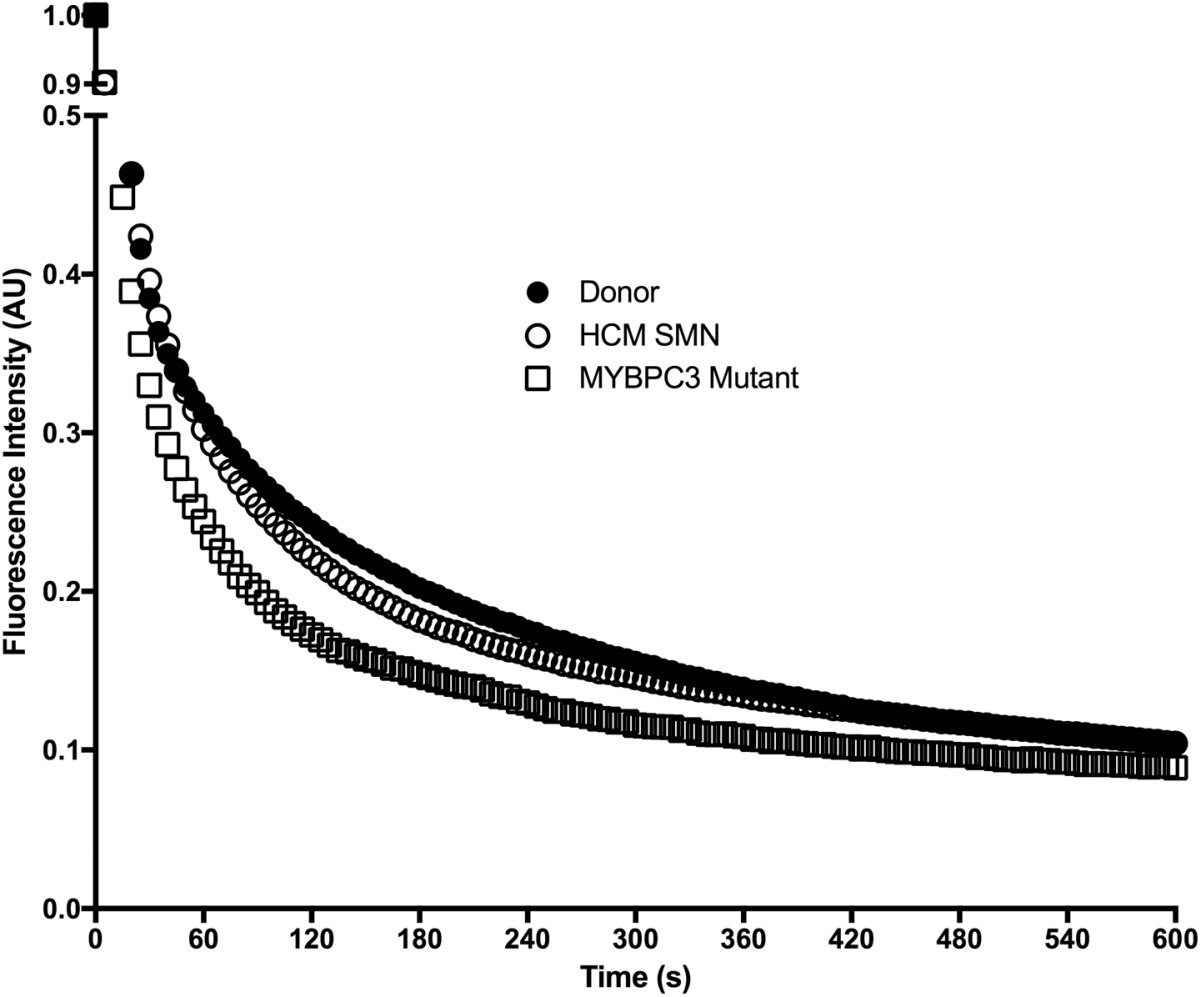
Representative fluorescence decay chase traces from donor, HCM_smn_ and *MYBPC3*_mut_ samples. Fibres were incubated in mATP and chased with ATP, and fluorescence intensity was monitored at 5 second intervals. Decay traces show an initial rapid phase, followed by a slow phase. The slow phase indicates the presence of myosin in the SRX. The decay in fluorescence intensity is nearly identical in donors (•) and HCM_smn_ (°). The fluorescence intensity from the *MYBPC3*_mut_ (☐) sample decays much more rapidly and exhibits a smaller slow phase. This represents a destabilised SRX. See S1 Text for the fit to these traces.

This is the first report to describe the presence of SRX myosin in human cardiac tissue. We used quantitative epifluorescence microscopy to measure single nucleotide turnovers in small bundles of permeabilised human LV cardiomyocytes. In these experiments, cardiomyocytes were incubated in relaxing buffer containing fluorescent mATP before being washed out with a relaxing solution containing ATP. The decay in fluorescent intensity of the cardiomyocytes was monitored at 5 s intervals. The reverse experiment (ATP incubation and mATP chase), has previously been demonstrated to have a symmetrical trace [21, 24], and therefore we did not perform this experiment in the present study. Fig 2 shows representative traces of data obtained from these experiments, with t = 0 representing the fluorescence intensity of the fibre immediately before washout with ATP.

**Fig 3:**
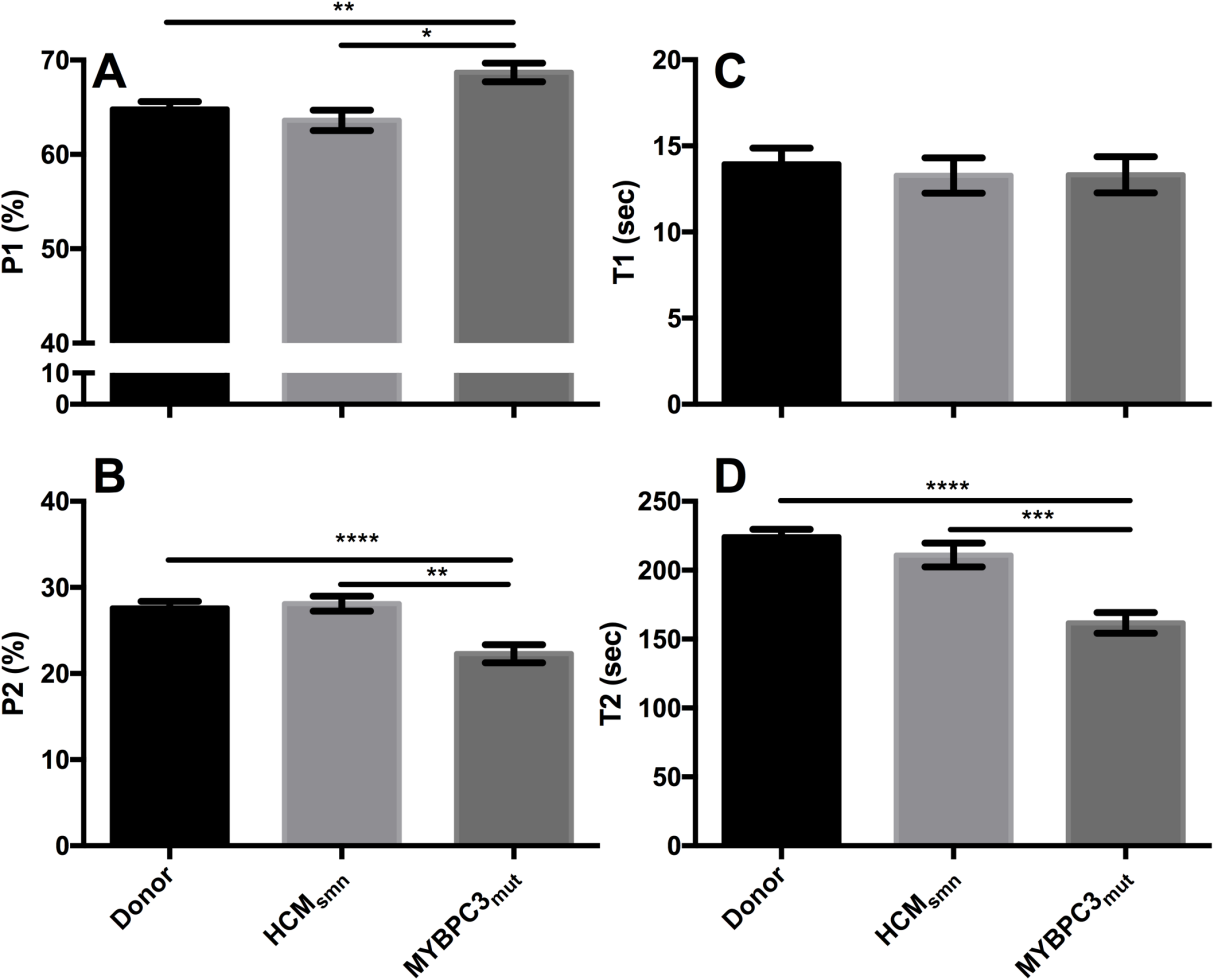
Summary of the variables derived from the two-state exponential in each of the studied groups. **A** The P1 is increased in *MYBPC3*_mut_ compared to the other groups, while the P2 is significantly reduced in *MYBPC3*_mut_ compared to donor and HCM_smn_ groups **(B). C** The lifetime of the rapid phase (T1) was not altered between groups. The lifetime of ATP turnover (T2) was also significantly decreased (**D**). Together these data show that mutations to *MYBPC3* significantly disturb the SRX. Data are expressed as mean ± SEM (see Table 2 for number of samples, technical repeats and statistical significance).

In skinned cardiomyocytes from donor LV, myosin in the SRX has an ATP turnover lifetime of 224 ± 6 s, while the P2 (the proportion of fluorescence attributed to the slow phase) is about 28% of the initial fluorescence (Fig 3). The fast phase, made up of multiple components including the DRX, exhibited a lifetime of 14.3 ± 0.9 s (T1), and contributed 65.1 ± 0.8% of the initial fluorescence. Calibration of this assay was carried out using glycerinated rabbit psoas muscle fibres, with results comparable to published data [21].

### Destabilised SRX in MYBPC3 mutants

Single myosin ATP turnover was measured in the LV of eight HCM patients containing mutations in *MYBPC3* (*MYBPC3*_mut_). Fig 2 shows representative ATP traces in permeabilised cardiomyocytes from this group, as well as the donor and HCM_smn_ groups. ATP turnover in both donor (closed circles) and HCM_smn_ (open circles) cardiomyocytes exhibit an initial rapid decay followed by a very slow decay in fluorescence intensity and, their traces are essentially co-incident. This slow decay is representative of a long-lived SRX in these cardiomyocytes. In contrast, the *MYBPC3*_mut_ trace decays more rapidly than both these groups (open squares), indicating a destabilisation of the SRX in these samples.

Fig 3 illustrates the average of the experimental fits obtained from each group, while Table 2 provides the data values. In Fig 3A there is a significant increase in the proportion of fluorescence attributed to the fast decay in fluorescence intensity (P1) of *MYBPC3*_mut_ compared to both donors (70 ± 1% vs. 65.1 ±0.8%, p < 0.01) and HCM_smn_ (70 ± 1% vs. 64 ± 1%, p < 0.05). This increase in P1 was coupled with a significant decrease in the proportion of myosin heads in the SRX (P2) in cardiomyocytes from *MYBPC3*_mut_ samples (Fig 3B) compared to both donor (22 ± 1% vs. 27.6 ± 0.7% respectively, p < 0.0001) and HCM_smn_ samples (22 ± 1% vs. 27.6 ± 0.7% respectively, p < 0.001). While the lifetime of the fast phase was unchanged between groups (Fig 3C), the lifetime of ATP turnover of myosin in the SRX (T2, Fig 3D) was significantly decreased in *MYBPC3*_mut_ compared to both donor (160 ± 7 s vs. 224 ± 6 s, p < 0.0001) and HCM_smn_ (160 ± 7 s vs. 211 ± 9 s, p < 0.001) samples. No significant differences were detected between the SRX of donor and HCM_smn_ groups.

**Table 2:** Comparison of the parameters of SRX experiments derived from age matched donor, *MYBPC3*_mut_ and HCM_smn_ tissue. The number of biological samples used in the study is represented by N. while the total number of technical repeats (n) is shown in parenthesis. P2 is an indicator of the number of myosin heads in the SRX. T2 is the lifetime of ATP turnover of these SRX myosin heads. Data are means ± SEMs.

	N (n)	P1 (%)	T1 (s)				P2 (%)		T2 (s)	
Donor	8 (40)	65.1 ± 0.8^b^	14.3 ± 0.9				27.6 ± 0.7^d^		224 ± 6^d^	
*MYBPC3*_mut_	8 (39)	70 ± 1	14 ± 1				22.3 ± 1		160 ± 7	
HCM_smn_	3 (16)	64 ± 1^a^	13 ± 1				28.1 ± 0.9^b^		211 ± 9^c^	

^a^ Indicates P<0.05 compared to *MYBPC3*_mut_

^b^ Indicates P<0.01 compared to *MYBPC3*_mut_

^c^ Indicates P<0.001 compared to *MYBPC3*_mut_

^d^ Indicates P<0.0001 compared to *MYBPC3*_mut_

### Destabilisation of the SRX irrespective of the nature of *MYBPC3* mutation

**Fig 4:**
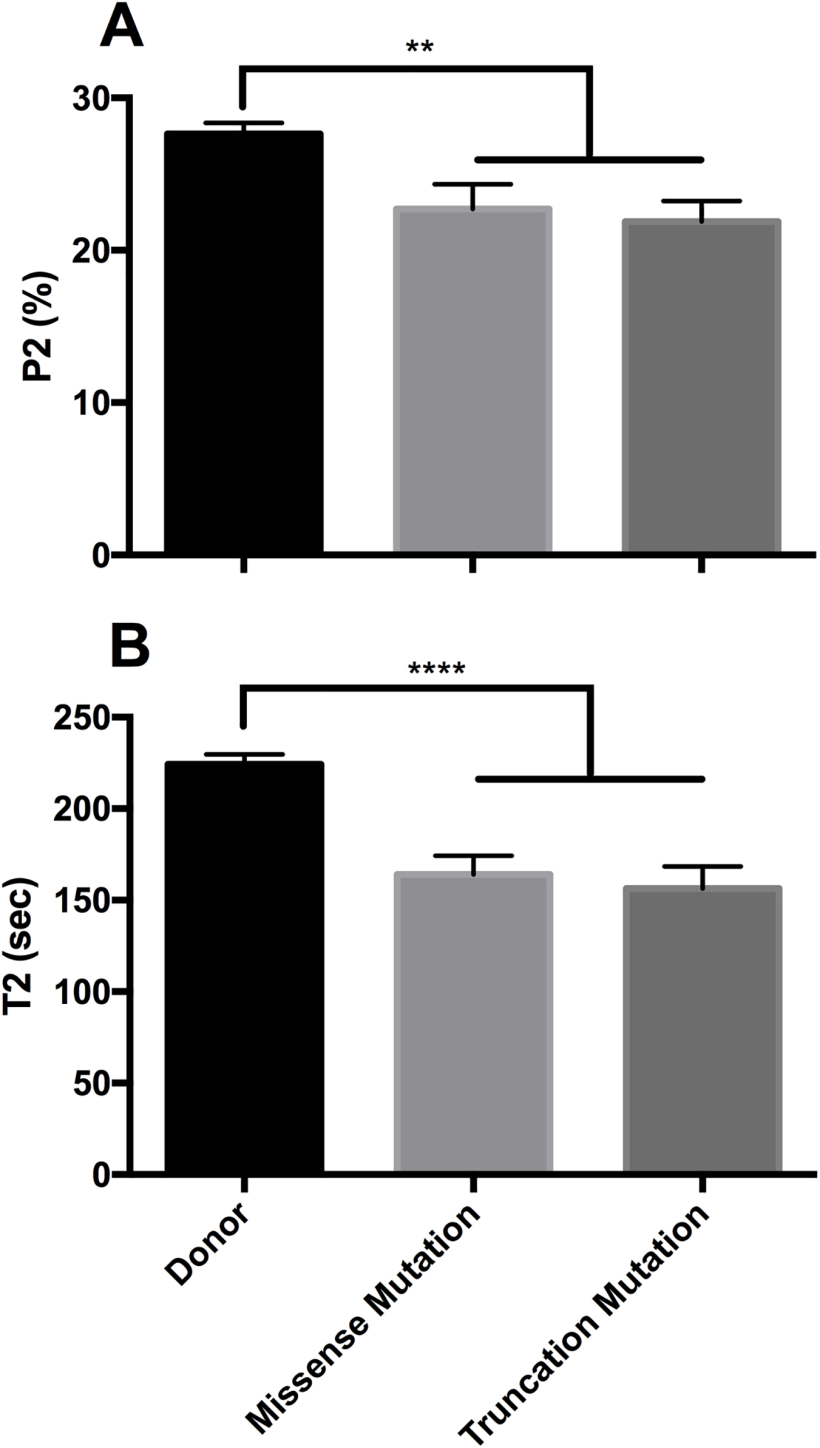
Reduction of the SRX is a common feature of *MYBPC3* mutations. The type of *MYBPC3* mutation (missense vs. truncations) does not alter the level of SRX destabilisation. (**A**) The proportion of myosin heads in the SRX (P2) and (B) the lifetime of ATP turnover of these SRX myosin are significantly decreased in both missense and truncating mutations compared to age matched donors. Data are expressed as mean ± SEM (n = 8 for donor and 4 for each mutation type; ** represents p < 0.01, **** represents p < 0.0001).

Mutations in *MYBPC3* may have two major effects on the protein product. Missense mutations result in the substitution of a single amino acid, whereas insertion/deletion or nonsense mutations often encode premature stop codons. Due to these stark structural differences, it is possible the SRX of these two groups display different levels of destabilisation. However, in this study, this was not the case, with both groups displaying a significantly reduced SRX compared to that of donor cardiomyocytes (Fig 4), and no differences between these groups.

### Expression of cMyBP-C and the SRX

**Fig 5:**
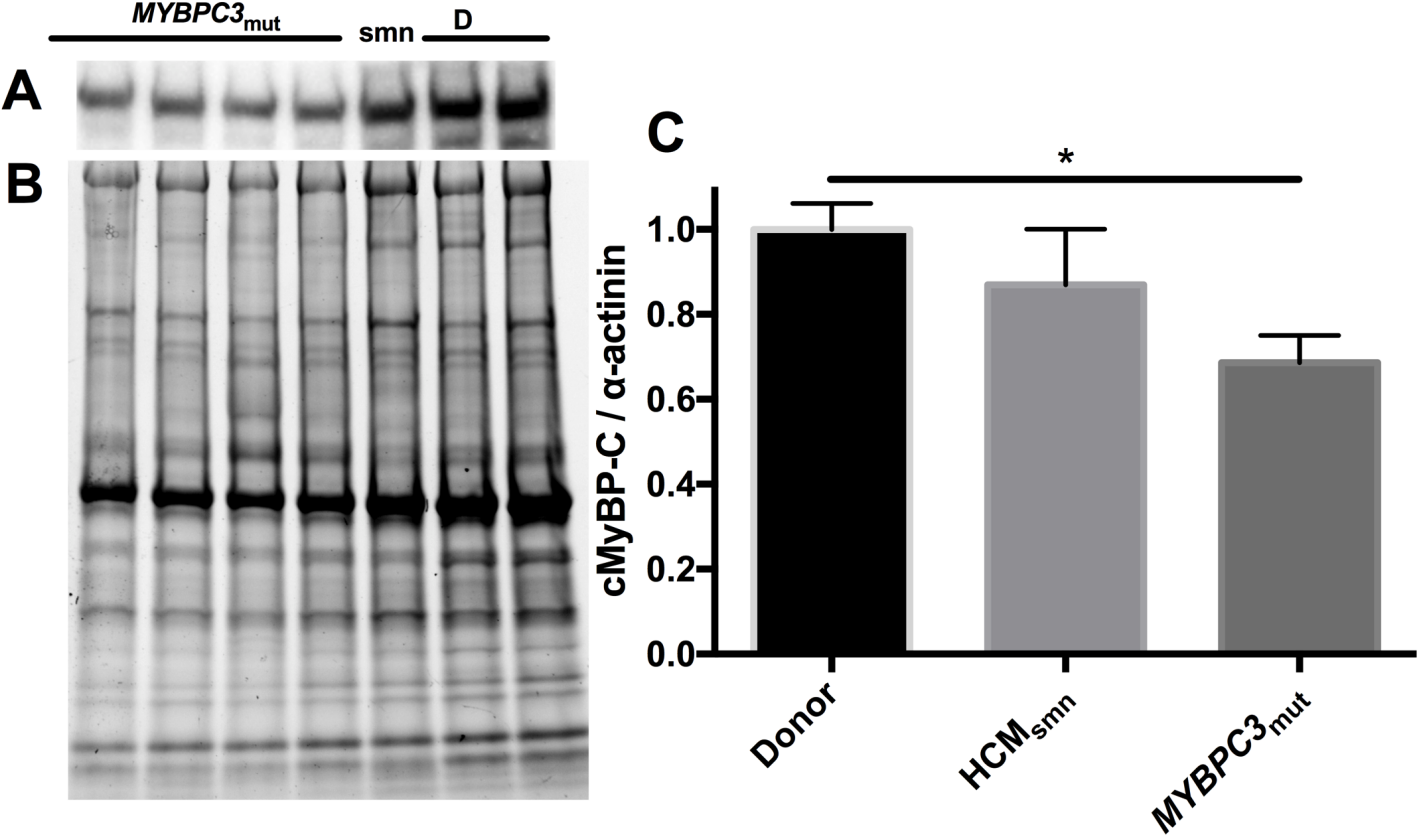
Expression of cMyBP-C in age matched donor (D), HCM_smn_ and *MYBPC3*_mut_ samples. Representative western blot (A) and corresponding stain-free blot (B) showing protein loadings. To account for changes in loading, cMyBP-C was normalised to α-actinin. (C) shows a significant decrease in the expression of cMyBP-C in *MYBPC3*_mut_ compared to age matched donors. HCM_smn_ did not achieve statistical significance, probably due to the small sample population. Each sample was run in duplicate. Data are expressed as mean ± SEM (number of patients = 4 for donors; = 2 for HCM_smn_; = 8 for *MYBPC3*_mut_; each sample was run in duplicate; * represents p < 0.05).

The expression of cMyBP-C in *MYBPC3*_mut_ was compared to donor and HCM_smn_ samples as reported previously [42] (Fig 5A and 5B). When data were grouped, *MYBPC3*_mut_ displayed decreased cMyBP-C content compared to donor hearts (p = 0.01). Fig 5C shows that in the *MYBPC3*_mut_ samples, cMyBP-C was expressed at roughly 68% of the donor levels, consistent with previously published results [42]. No differences in cMyBP-C expression were observed between donor and HCM_smn_ groups.

**Fig 6:**
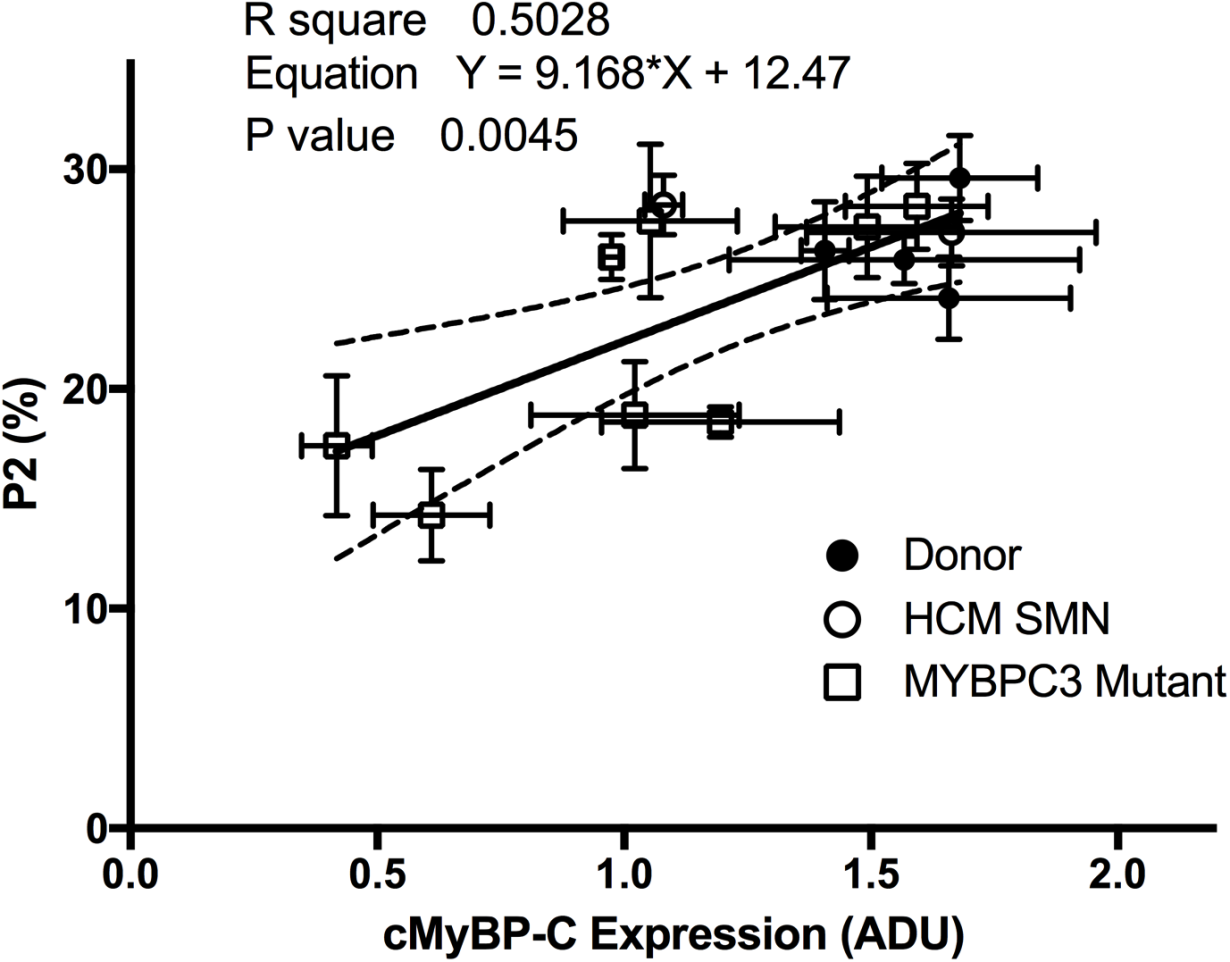
cMyBP-C expression plotted against P2. The western blot- determined expression level of each sample (normalised to α-actinin) was plotted against its respective P2 value, revealing a positive correlation between these two values. This suggests that the cMyBP-C tethers myosin heads to the thick filament. The absence of cMyBP-C destabilises the SRX. Interestingly the theoretical Y-intercept is similar to the P2 observed in mice lacking cMyBP-C. ADU represents “arbitrary density units”, as determined from normalization of cMyBP-C to α-actinin.

To determine if the expression levels of cMyBP-C may control the level of disruption of the SRX, the P2 of individual samples was plotted against cMyBP-C expression level (Fig 6). There was a positive correlation between these two parameters (r^2^ = 0.57, p < 0.01). Samples with the lowest expression levels of cMyBP-C displayed the lowest P2 of the SRX and those with no change in the P2 generally did not exhibit haploinsufficiency.

### Endogenous phosphorylation of sarcomeric proteins

**Fig 7:**
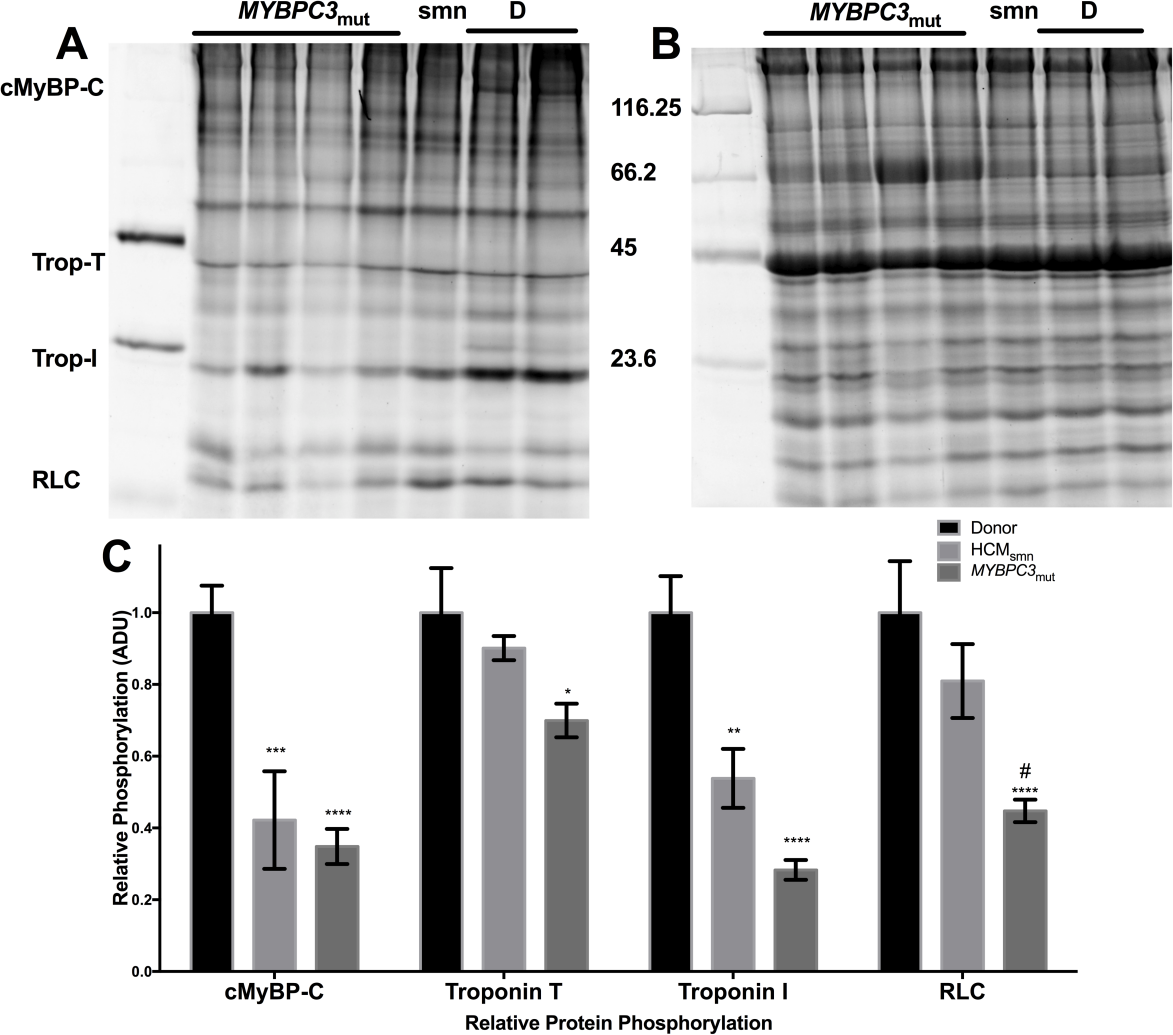
Phosphorylation of key sarcomeric proteins were measured by ProQ diamond staining, A. Panel A also shows the location of the sarcomeric proteins measured in this study. Panel B shows the same gel stained with Sypro Ruby, with the molecular weights of the marker shown to the left. The relative phosphorylation of each protein is shown in panel C, normalised to the donor group. Data are expressed as mean ± SEM (n = 4, 2 and 8 for donor, HCM_smn_ and *MYBPC3*_mut_, each sample was run in duplicate and data pooled respectively; *, **, *** and **** represent p < 0.05, 0.01, 0.001 and 0.0001 compared to donor; # represents p < 0.05 compared to HCM_smn_). The molecular weight marker used is the Peppermint Stick Marker, supplied with ProQ Diamond kit.

The endogenous phosphorylation status of key sarcomeric proteins (cMyBP-C, troponin-T, troponin-I and the regulatory light chain of myosin) was quantified by ProQ Diamond staining (Fig 7A). The density of each ProQ band was normalised to the density of α-actinin stained by Sypro Ruby (Fig 7B). The results for each group were averaged and normalised to the age-matched donor group. The normalised levels of phosphorylation for each sarcomeric protein are shown in Fig 7C.

Phosphorylation of cMyBP-C was reduced by roughly 60% in both HCM_smn_ and *MYBPC3*_mut_ (p < 0.001 and 0.0001, respectively). Troponin-I phosphorylation was also significantly reduced in both HCM_smn_ and *MYBPC3*_mut_ compared to donor samples (p < 0.01 and 0.0001, respectively). The phosphorylation of troponin-T was also reduced in both disease groups, however only *MYBPC3*_mut_ reached significance compared to the donors (p < 0.05). Interestingly, the regulatory light chain (RLC) phosphorylation of *MYBPC3*_mut_ was significantly reduced compared to both donors and HCM_smn_ (p <0.0001 and 0.05, respectively). The RLC phosphorylation of HCM_smn_ was not significantly reduced compared to donor samples (p = 0.4).

## Discussion

### The super relaxed (SRX) state in human myocardium

The SRX has been reported in a variety of muscles and organisms [20–24], but this is the first report of the SRX in human tissue, and confirms structural data that reported the presence of the interacting heads motif in human cardiac thick filaments [30]. The population of the SRX (P2), and its lifetime (T2) are both indicators of stability of the SRX. While the P2 is similar between rabbit and donor human cardiac muscle, the T2s vary. The T2 of human cardiac muscle is longer than that of the rabbit (~220s vs. ~140s) [21], possibly reflecting the difference in heart rate (~80 vs 150 bpm) of the two species. Supporting this hypothesis is our previous finding that mice, with a faster heart rate (~450bpm), have an even shorter T2 of ~100s [22].

The SRX provides a state to “park” myosin heads that are not being used into a very economical state. In cardiac cells, it also provides a level of thick filament regulation of active muscle, in addition to the well-documented thin filament control [46]. As myosin heads in the SRX are sterically restrained from interaction with actin, a decrease in the proportion of myosin in the SRX and concomitant increase in the DRX would result in more myosin heads capable of actin interaction. This increased myosin:actin interaction may contribute to the hyperdynamic systolic function, typically observed in HCM.

### Disruption of the super-relaxed state in LV from *MYBPC3* mutants

The major finding of this study is that mutations in *MYBPC3* in humans with HCM are associated with a disrupted SRX. There was a 20% reduction in the P2 (Fig 3C) of *MYBPC3*_mut_ compared to donor cardiomyocytes. Through a competition assay, we have previously shown that due to non-specific binding of the mATP to the cardiomyocytes, the actual number of myosin heads in the SRX is approximately double that of P2 [22]. This corresponds to 56% of myosin heads in the SRX in donor muscle, which is reduced to 44% in *MYBPC3*_mut_ samples (Table 2). Therefore, about 10% of myosin heads are lost from the SRX in cardiomyocytes from *MYBPC3*_mut_ samples. Coupled with the increase in P1 for these samples, we believe this constitutes strong evidence that a fraction of myosin heads in these mutant hearts are released from the thick filament, and enter into the DRX state. This destabilisation is less severe than we reported for homozygous knockout mice that lacked cMyBP-C [22]. This finding is consistent with our hypothesis that cMyBP-C stabilises the SRX, given cMyBP-C is still expressed in HCM_smn_.

Our results agree with recent findings that cMyBP-C promotes the “OFF” state of thick filaments, which is analogous to the SRX [47]. A number of correlations have been drawn between the SRX, a functional measurement, and the structural state known as the OFF state [23, 24]. These have generally supported the hypothesis that the myosin heads in the SRX are also in the OFF state. However, the possibility of a well-structured state, which do not have inhibited ATPase activity remains a possibility. Given each group was imaged under identical image settings, we do not anticipate that potential scattering of the 395nm excitation would alter our interpretation of the results.

A subset of data was also fit to a sum of three exponential processes. This yielded a fast phase (P1 ~45%, T1 ~20 s), an intermediate phase (P2 ~ 32%, T2 ~80s), and a longer-lived state (P3 ~ 17%, T3 ~ 550 s). However, a comparison of the absolute sum of squares suggested that this three-exponential fit was not justified. Regardless of the number of exponents used, our interpretation that *MYBPC3* mutations reduced the SRX remains unchanged.

Interestingly, there was also a significant decrease in the lifetime of ATP turnover (T2) in the *MYBPC3*_*mut*_ compared to age-matched donors (Fig 3B). This contrasts with our previous report in mice lacking cMyBP-C, a decrease only in the P2 [22]. It is possible that alterations to cMyBP-C (due to *MYBPC3* mutation) affect the catalytic domain of myosin heavy chain, altering its ATPase activity in this SRX, which has previously been hypothesised [48, 49]. This may occur due to a weakened interacting heads motif within the C zone. Together with the reduced P2, the data suggest that *MYBPC3*_mut_ exhibit a further loss of economy, whereby there is a reduced number of myosin heads in the energy conserving SRX (P2) but also the remaining heads in this state hydrolyse and turnover nucleotides more rapidly. It is important to note that while sarcomere length was not controlled in this study, it has previously been shown that the SRX is not altered at different sarcomere lengths under relaxing conditions [21, 24]. Therefore, it is unlikely that this contributes significantly to the findings presented here. Additionally, these experiments were carried out using septal tissue from the LV side for HCM samples and LV free-wall samples for donor samples. Donor LV has previously been used as controls to HCM septal tissue [42, 50], and as there were no changes in the SRX of donor LV and HCM_smn,_ we do not believe this significantly alters our interpretation.

Two *MYBPC3*_mut_ samples carried secondary mutations. M21 had two *MYBPC3* mutations (E542Q, V375E). This sample exhibited a P2 of 17 ± 3%, and a T2 of 201 ± 27 s. M13 carried a *MYBPC3* mutation (D770N) and a *MYL3* mutation (E143K), and both the P2 (14 ± 2%), and the T2 (138 ± 30 s) were severely reduced. Interestingly, *MYL3* encodes the ventricular isoform of the essential light chain (ELC) of myosin, which is predicted to play a role in the interacting heads motif, with the ELC of a blocked myosin head interacting with the neighbouring free myosin head [51]. Therefore, the combination of these two mutations may cause a compound heterozygous effect on the SRX state. SRX data for individual samples is shown in Table A in S1 Text.

The availability of LV tissue from HCM_smn_ patients proved invaluable to this study and served as a negative control for the effect of cardiac hypertrophy and fibrosis in the study. Our results show that the SRX of the *MYBPC3*_mut_ was also severely diminished compared to the HCM_smn_ (Fig 3). Taken together, these results specifically implicate mutations in *MYBPC3* rather than HCM as the source for this disrupted SRX.

**Fig 8:**
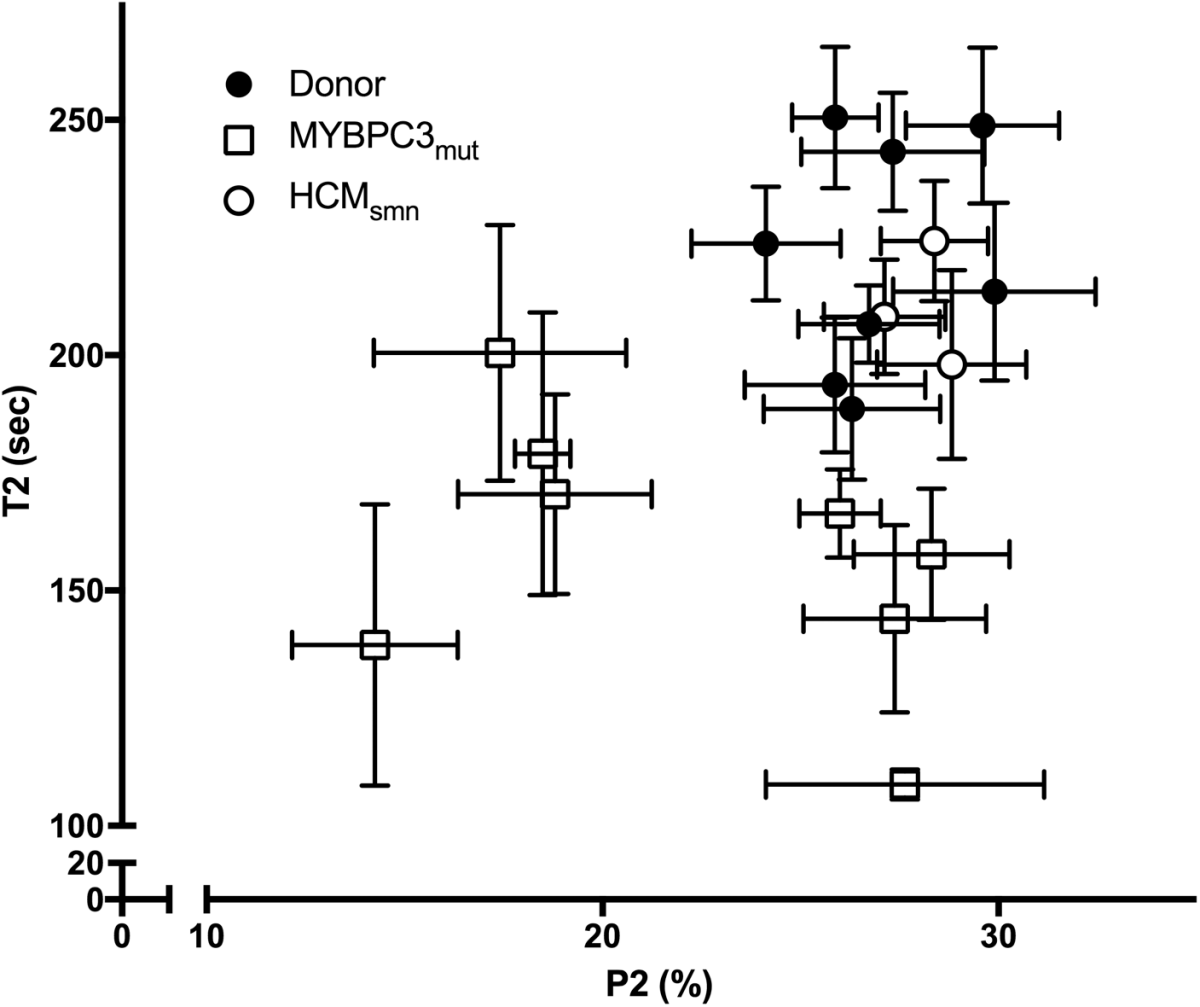
Plot of T2 (s) vs. P2 (%) for each sample used in this study. Donors (•) and HCM_smn_ (°) cluster towards the top right of the graph. However, the *MYBPC3*_mut_ (☐) samples have a wide spread of values, with none in the upper right quadrant. This shows destabilisation of the SRX in all *MYBPC3*_mut_ *samples*, irrespective of the type of mutation or expression level of cMyBP-C.

Fig 8 plots P2 versus T2 for each sample, both of which reflect the stability of the SRX. This plot demonstrates that donor and the negative control HCM_smn_ samples cluster in the top right of the graph, while *MYBPC3*_mut_ samples are widely spread. These *MYBPC3*_mut_ samples do appear to cluster into two groups, one of which shows a reduced P2, and the other a reduced T2. It is possible that this reflects two different mechanisms by which *MYBPC3* mutations affect the SRX, however this remains speculative. While every *MYBPC3*_mut_ sample used in this study displayed a destabilised SRX, in some samples the destabilisation was more evident in P2 while in others it was more evident in T2. This suggests that destabilisation of the SRX may be a common feature of HCM related *MYBPC3* mutations. A larger cohort would be required to confirm these findings.

### Correlation of the SRX and expression of cMyBP-C

Many mutations in *MYBPC3* lead to sub-stoichiometric quantities of cMyBP-C termed haploinsufficiency [42, 52]. Reduced amounts of cMyBP-C may cause a decrease in the population of myosin in the SRX by decreasing the physical restraint on the myosin heads. This shift of myosin heads to the DRX would result in greater active forces and higher ATP demands and could contribute to hypertrophic remodelling [53]. Western blots measured the cMyBP-C content in each of the groups. When data from each group were pooled, *MYBPC3* mutants expressed ~68% of the cMyBP-C compared to age-matched donors (Fig 5C, P < 0.05). This reduced expression is consistent with previously published results [42, 54]. No differences in cMyBP-C expression were measured between HCM_smn_ and donor hearts, also consistent with previous work.

While the pooled data suggests haploinsufficiency, not every *MYBPC3*_mut_ sample exhibited this effect. This sample-to-sample variability has previously been reported [55, 56], and likely depends on the nature of the specific mutation. Thus, we plotted the expression profiles of each mutant and donor against their respective P2 values (Fig 6). This revealed a positive correlation between these parameters (r^2^ = 0.57, p < 0.01). Samples with a diminished P2 generally exhibited lower expression of cMyBP-C. Interestingly, the theoretical Y-intercept of the linear regression fit (i.e. no cMyBP-C) is P2 = 12%, similar to that observed in mice completely lacking the protein altogether (P2 = 8.5%) [22]. These results suggest that cMyBP-C content is related to the number of myosin heads in the SRX, and is consistent with our hypothesis that cMyBP-C is required to maintain the SRX.

A recent report used cultured neonatal cMyBP-C knockout cardiomyocytes in a specialised silicone mould to produce genetically engineered “heart tissue”. These neonatal cardiomyocytes were transfected with varying levels of human cMyBP-C. When cMyBP-C levels were lower than 73% of the wild-type level, contractile function was unchanged compared to knockout myocytes, while higher expression levels more closely resembled wild-type tissue [57]. In our study, we found if cMyBP-C expression was below this 73% mark in all samples that exhibited a loss of P2 (Fig 6). While this was the case, it is important to note that all *MYBPC3*_mut_ exhibited a destabilised SRX with reduced values of P2, T2 or both (Fig 8), again suggesting a universal effect on the SRX. These results show that while haploinsufficiency plays an important role, it cannot be solely attributed to the reduced SRX.

### Phosphorylation of sarcomeric proteins

Phosphorylation of sarcomeric proteins was measured using ProQ Diamond staining (Fig 7). cMyBP-C phosphorylation was significantly reduced in both *MYBPC3*_mut_ and HCM_smn_ compared to donors (p < 0.0001 and p < 0.001, respectively). Similarly, the troponin I phosphorylation was reduced in both HCM groups compared to the donor group (p < 0.01 for HCM_smn_, p < 0.0001 for *MYBPC3*_mut_). These altered phosphorylation levels have previously been reported [42], and our results validate these findings. Presumably, dephosphorylation of cMyBP-C would provide greater stability to the SRX, based on previous X-ray diffraction studies [58–60]. Thus, we propose that the alterations to cMyBP-C structure and expression outweigh the effects of this dephosphorylation. Additionally, Colson, et al. reported no change in the I_1,1_/I_1,0_ ratio in skinned papillary muscles containing wild-type or phospho-ablated cTnI [60]. While cTnI dephosphorylation is associated with diastolic dysfunction and increased calcium sensitivity, this suggests it plays no role in modulation of the SRX.

Interestingly, the level of phosphorylation of the regulatory light chain (RLC) in *MYBPC3*_mut_ samples was reduced compared to both donor and HCM_smn_ samples (p < 0.0001 and P < 0.05, respectively). Decreased RLC phosphorylation stabilises the SRX [24, 61, 62], however extensive studies are required to determine if this difference has any physiological role. We note that the phosphorylation of troponin T was significantly reduced compared to donors only in *MYBPC3*_mut_ samples. It is possible that dephosphorylated TnT destabilises the ordering of the thick filament, but this relationship has not been reported previously. Additionally, it is possible that the use of β-blockers and calcium channel blockers in these two groups are the source of decreased sarcomeric protein phosphorylation. However, the prescribed medications were similar in both HCM_smn_ and *MYBPC3*_mut_ cohorts, and thus should not confound the interesting findings between these two groups (S1 and S2 Tables).

### Possible consequences of a reduction in the SRX

In this study, we have shown that mutations in *MYBPC3* are associated with a shift of myosin heads from the SRX into the DRX. Although the thick filament structure of *MYBPC3*_mut_ has not directly been studied, myosin heads can be expected to display greater disorder along the thick filament. This is supported by the finding that mice lacking cMyBP-C exhibit thick filament disorder [33]. Future studies are required to determine the degree of myosin head order in these samples. This structural change to the thick filament could significantly alter both cardiac contractility and energetics (for a quantitative discussion, see [21]). Quantifying the SRX in living cardiomyocytes is much more difficult. However, recent work using time resolved X-ray diffraction on intact rat trabeculae showed that the helical ordering of myosin heads, present in resting muscle, is reduced when muscle contraction occurs at longer sarcomere lengths [63]. This suggest that the SRX is likely present in intact muscle.

Depending on the mutation, it is possible that specific mutations in cMyBP-C reduce its affinity for myosin. Alternatively, these mutations may result in a reduced content of cMyBP-C in the thick filament. Both of these mechanisms would reduce the strength of the cMyBP-C:myosin interaction and untether myosin heads from the thick filament. This increase of myosins in the DRX would result in a greater number of myosin heads available for contraction and thus may be partially responsible for the hypercontractile phenotype observed in patients with these mutations [48].

Destabilisation of the SRX in hearts containing *MYBPC3* mutations would also result in increased energy utilisation within the sarcomere. Our results showed fewer myosin heads in the SRX, but also that the heads remaining in the SRX exhibit a faster ATPase rate. In human LV, myosin heads in the DRX state have an ATPase activity more than 7-fold faster than in the SRX [64]. The ATPase activity of the DRX is assumed to be equal to the ATPase of purified β-cardiac myosin (1 ATP every 30 seconds) [24, 64]. In cardiomyocytes from *MYBPC3*_mut_ samples, the ATP turnover of myosin heads that remain in the SRX is increased by nearly 30% (Fig 3D). Positron emission tomography (PET) and magnetic resonance imaging (MRI) of pre-hypertrophic patients with *MYBPC3* and *MYH7* mutations demonstrated decreased myocardial external efficiency compared to healthy patients [65]. Similarly, the PCr/ATP ratio in HCM patients with *MYBPC3* mutations was decreased compared to non-failing subjects [66], suggesting altered cardiac energetics in these patients.

The faster turnover of ATP in *MYBPC3*_mut_ cardiomyocytes would increase energy demands on the heart and possibly contribute to the remodelling seen in HCM [53]. This concept has been suggested previously [67] with respect to myosin heavy chain mutations in HCM. Herein, we were able to demonstrate that this hypothesis may hold true for mutations in *MYBPC3*.

Proving the hypothesis that a destabilised SRX contributes to the signalling that initiates hypertrophic remodelling may prove difficult, but the recent discovery of the small molecule MYK-461 may provide some insights. MYK-461 suppresses myosin ATPase activity, and mice with HCM-associated mutations showed blunted development of HCM following administration of this drug [68]. It is possible that this, and other molecules may be used to directly target myosin in the SRX, although this remains to be shown.

As discussed previously, a possible limitation of this study is that the SRX has been studied in glycerinated muscle fibres rather than intact cardiomyocytes. This step would be very difficult, due to the use of cryopreserved tissue, and is not possible with the current experimental protocols. Additionally, the use of TIRF microscopy to directly image nucleotide turnover within the A-band could provide improved signal to noise ratio. However, this experimental apparatus was not available to us.

In summary, this report has extended our previous work [22] with a mouse model to human patients with *MYBPC3* mutations. We compared the SRX of skinned LV cardiomyocytes from these patients with age-matched donors and HCM patients where there was no known mutation in sarcomeric genes. Results show a significant decrease in the proportion of myosin heads in the SRX of hearts containing *MYBPC3* mutations. Interestingly, the lifetime of ATP turnover was also reduced in these hearts, an effect not observed in the mouse model. This would result in an additional loss of energetic economy in these hearts, and may be a contribute to the development of cardiac hypertrophy in these patients, as suggested previously[53].

Work is underway to determine the mechanism by which cMyBP-C regulates the SRX. Future studies will also determine if mutations that affect myosin heavy chain result in a similar effect on the SRX. Studies using more patients and a wider variety of *MYBPC3* mutations would also determine if these effects are similar in any pathological variant of *MYBPC3* or if mutations specific differences exist.

## Supporting information

S1 Text Non-linear regression fits corresponding to Fig 2 of main text. **Figure A -**A. Representative DIC image of a muscle fibre used in this study, and B. the corresponding fluorescent image following incubation with mATP. **Figure B—**Schematic describing the glycerinating process and the flow cell used in the experiments. Small fragments of muscle were collected under liquid nitrogen and processed as described in the methods. Small bindles of muscle fibres were dissected in glycerinating solution and immobilized to a glass coverslip. **Table A-** Individual SRX data for each sample used in this study.
(DOCX)

S1 Table Extended patient data for *MYBPC3*_mut_ samples used in this study. (XLSX)

S2 Table Extended patient data for HCM_smn_ samples used in this study. (XLSX)
