# Supplementary material for: MYBPC3 mutations are associated with a reduced super-relaxed state in patients with hypertrophic cardiomyopathy
Source: PLoS One. 2017 Jun 28;12(6):e0180064. doi: 10.1371/journal.pone.0180064 (PMC5489194; doi:10.1371/journal.pone.0180064)
Supplement: S1 Text — Figure A -A. Representative DIC image of a muscle fibre used in this study, and B. the corresponding fluorescent image following incubation with mATP. Figure B—Schematic describing the glycerinating process and the flow cell used in the experiments. Small fragments of muscle were collected under liquid nitrogen and processed as described in the methods. Small bindles of muscle fibres were dissected in glycerinating solution and immobilized to a glass coverslip. Table A- Individual SRX data for each sample used in this study. (DOCX) [file pone.0180064.s001.docx]

**Supplemental Material**

**MYBPC3 Mutations are Associated with a Reduced Super-Relaxed State in Patients with Hypertrophic Cardiomyopathy**

James W. McNamara^1,2*^, Amy Li^3^, Sean Lal^1^, J. Martijn Bos^4^, Samantha P. Harris^5^, Jolanda van der Velden^6^, Michael J. Ackerman^4^, Roger Cooke^7^, Cristobal G. dos Remedios^1^

^1^Discipline of Anatomy & Histology, Bosch Institute, University of Sydney, NSW 2006, Australia.

^2^Division of Cardiovascular Health and Disease, University of Cincinnati College of Medicine, Cincinnati, OH 45267, United States.

^3^Department of Molecular Physiology and Biophysics, University of Vermont, Burlington, VT 05405, USA.

^4^Windland Smith Rice Sudden Death Genomics Laboratory, Mayo Clinic, Rochester, MN, 55905

^5^Department of Cellular and Molecular Medicine, University of Arizona, Tucson, AZ 85721, USA.

^6^Department of Physiology, Institute for Cardiovascular Research, VU University Medical Center, De Boelelaan 1117, 1081HC Amsterdam, The Netherlands.

^7^Department of Biochemistry and Biophysics, Cardiovascular Research Institute, University of California, San Francisco, CA 94143-22240, USA.

^*^Corresponding author at: ^2^Division of Cardiovascular Health and Disease, University of Cincinnati College of Medicine, Cincinnati, OH 45267, United States. Email address: mcnamajw@ucmail.uc.edu

**S1 Text A: Non-Linear Regression Fits to Figure 2**

Donor (closed circles): P1 = 63.8 ± 0.3%; T1 = 13.5 ± 0.2 s; P2 = 27.65 ± 0.3%; T2 = 218 ± 4 s

HCM_smn_ (open circles): P1 = 65.4 ± 0.7%; T1 = 14.8 ± 0.4 s; P2 = 25.5 ± 0.6%; T2 = 191 ± 8 s

*MYBPC3*_mut_ (open squares): P1 = 71.6 ± 0.9%; T1 = 13.3 ± 0.4s; P2 = 20.2 ± 0.8%; T2 = 167 ± 11 s

**S1 Fig A**

S1 Figure A: **A** Representative DIC image of a fibre preparation used, **B** the corresponding fibre following incubation with mATP.

**S1 Fig B
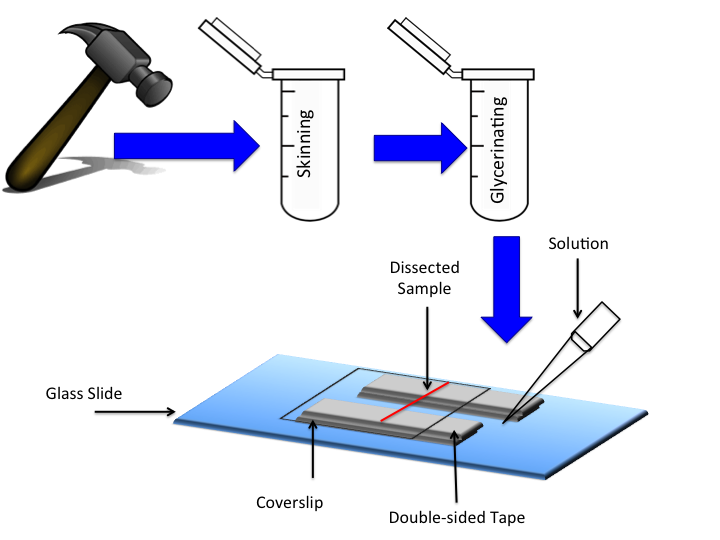
**

S1 Fig B: Diagram of the apparatus used to measure the SRX state.

**S1 Table A: Individual Sample SRX data**

|  |  |  | P2 (%) | | T2 (s) | |  |
| --- | --- | --- | --- | --- | --- | --- | --- |
|  | Sample Code | Mutation(s) | Mean | SEM | Mean | SEM | n |
| *MYBPC3mut* | M21 | V365E; E542Q | 17 | 3 | 201 | 27 | 4 |
|  | M13 | D770N; E143K (*MYL3*) | 14 | 2 | 138 | 30 | 4 |
|  | M23 | E542Q | 26 | 1 | 166 | 9 | 6 |
|  | M5 | E461X | 19 | 2 | 171 | 21 | 6 |
|  | M7 | V1063 fs/63 | 28 | 4 | 109 | 3 | 4 |
|  | M24 | L527 fs/3 | 18.5 | 0.7 | 179 | 30 | 5 |
|  | M10 | Q791 fs/40 | 27 | 2 | 144 | 20 | 5 |
|  | M2 | R1073W | 28 | 2 | 158 | 14 | 5 |
| Donors | 3.164 - 61 | None | 26 | 2 | 189 | 15 | 6 |
|  | 2.158 - 17 | None | 26 | 1 | 251 | 15 | 5 |
|  | 6.008 | None | 24 | 2 | 224 | 12 | 5 |
|  | 8.01 | None | 30 | 2 | 249 | 17 | 5 |
|  | 7.028 | None | 30 | 3 | 214 | 19 | 4 |
|  | 6.076 | None | 27 | 2 | 243 | 13 | 7 |
|  | 5.138 | None | 26 | 2 | 194 | 14 | 6 |
|  | 3.168 | None | 27 | 2 | 207 | 8 | 5 |
| HCM_smn_ | A48 | None | 28 | 1 | 224 | 13 | 6 |
|  | A45 | None | 27 | 2 | 208 | 12 | 5 |
|  | A22 | None | 29 | 2 | 198 | 20 | 5 |
